# Discordant conceptualisations of eating disorder recovery and their influence on the construct of terminality

**DOI:** 10.1186/s40337-024-01016-w

**Published:** 2024-06-03

**Authors:** Rosiel Elwyn, Marissa Adams, Sam L. Sharpe, Scout Silverstein, Andrea LaMarre, James Downs, C. Blair Burnette

**Affiliations:** 1 Neuroscience and psychiatry, Thompson Institute, Birtinya, QLD Australia; 2 University of the Sunshine Coast, Birtinya, QLD Australia; 3 Independent Researcher, Nashville, TN USA; 4 Fighting Eating Disorders in Underrepresented Populations (FEDUP, Collective), West Palm Beach, FL USA; 5 Project HEAL, New York, NY USA; 6 Independent Researcher, Halifax, NS Canada; 7 Independent Researcher, Cardiff, Wales UK; 8 Department of Psychology, Michigan State University, Lansing, MI USA

**Keywords:** Longstanding eating disorder, Severe and enduring eating disorders, Iatrogenic harm, Futility, Terminality, Terminal anorexia nervosa, Outcome, Recovery, Lived experience

## Abstract

Eating disorders (EDs) are complex, multifaceted conditions that significantly impact quality-of-life, often co-occur with multiple medical and psychiatric diagnoses, and are associated with a high risk of medical sequelae and mortality. Fortunately, many people recover even after decades of illness, although there are different conceptualisations of recovery and understandings of how recovery is experienced. Differences in these conceptualisations influence categorisations of ED experiences (e.g., longstanding vs. short-duration EDs), prognoses, recommended treatment pathways, and research into treatment outcomes. Within recent years, the proposal of a ‘terminal’ illness stage for a subset of individuals with anorexia nervosa and arguments for the prescription of end-of-life pathways for such individuals has ignited debate. Semantic choices are influential in ED care, and it is critical to consider how conceptualisations of illness and recovery and power dynamics influence outcomes and the ED ‘staging’ discourse. Conceptually, ‘terminality’ interrelates with understandings of recovery, efficacy of available treatments, iatrogenic harm, and complex co-occurring diagnoses, as well as the functions of an individual’s eating disorder, and the personal and symbolic meanings an individual may hold regarding suffering, self-starvation, death, health and life. Our authorship represents a wide range of lived and living experiences of EDs, treatment, and recovery, ranging from longstanding and severe EDs that may meet descriptors of a ‘terminal’ ED to a variety of definitions of ‘recovery’. Our experiences have given rise to a shared motivation to analyse how existing discourses of terminality and recovery, as found in existing research literature and policy, may shape the conceptualisations, beliefs, and actions of individuals with EDs and the healthcare systems that seek to serve them.

## Introduction

Within the field of eating disorders (EDs), there are different understandings of EDs regarding aetiology, nosological constructs, and optimal treatment approaches across clinicians, researchers, and the people with lived ED experience (including those who are also clinicians and researchers). Variations in theory, policy, clinical practice, and widespread contradictions and paradoxes in ED literature may contribute to the lack of definitional consensus on ED recovery. Despite these challenges of achieving consensus on key components of ED recovery, recent research efforts have focused on exploring the potential benefits of defining severe and enduring EDs (SE-EDs) or severe and enduring anorexia nervosa (SE-AN), hereafter referred to as longstanding EDs^1^ [1–3]. There is also increased discussion in the literature about the concept of ED treatment futility in longstanding EDs (usually specific to anorexia nervosa), where additional attempts at treatment are suggested to have little benefit and/or ‘full’ recovery is unlikely.

Recently, a set of criteria was created, attempting to conceptualise ‘terminal anorexia nervosa’ [4–6], which has been widely critiqued, including by individuals with lived experience [7–18], and other clinician-researchers [19–25]. We value the contributions of these diverse perspectives and intend to contribute to these topics by considering how the conceptualisation of terminality^2^ is informed by corresponding conceptualisations of recovery, treatment response, and quality-of-life. This article also critically analyses the medical lens through which these concepts are understood and explores how nosological, etiological, iatrogenic, and ethical factors influence conceptualisations of ED recovery, longstanding EDs, treatment futility, and ED ‘terminality’.

## Positionality

First, we wish to acknowledge our positionalities, not as a check-box exercise to disclaim our biases but to situate ourselves and consider how our lenses have inevitably shaped our perspectives on terminality and recovery. The authors of this paper have lived or living experience with EDs and are also clinicians, advocates, activists, nonprofit professionals, and researchers in the fields of EDs, public health, psychology, neuroscience, and biology. We have navigated our experiences with EDs and within these professional settings while holding a variety of identities that are historically oppressed and excluded from mainstream acceptance of who develops EDs and/or who is qualified to work in a clinical and/or research capacity with EDs. Our experiences as professionals with a range of different EDs inform this paper. We also occupy various other positions; some are LGBTQA+, intersex, autistic, and disabled. We recognise there are many experiences we cannot speak to and recognise the heterogeneity of identities within our collective. We are all from Western countries (Australia, the USA, Canada, and the UK), which impacts our personal positionalities and which systems of ED care we can most knowledgeably discuss. We did not write this paper to argue for one singular way of conceptualising or practising; indeed, there are differences in how we (as individuals) envision the topics we address. We collectively drew on our perspectives to develop a paper that offers opportunities to think through terminality, treatment futility, and recovery rather than suggest a singular solution.

## Defining recovery: elusive standards and variable contexts

If providers, loved ones, and people with living or lived experience *all* hold varying ideas about what recovery ‘is’ or could be, making decisions about the possibility of and/or capacity for recovery becomes challenging. A person may be rendered ‘unrecoverable’ by clinical discourses that write their future based on preconceived notions about the kinds of lives they can or cannot live [26, 27]. Remarkably, discussions about what recovery is – and how to ‘get there’ – remain curiously absent from many discussions about terminality. This absence is particularly notable given that there is no singular definition of recovery, nor complete agreement amongst individuals with lived experience, clinicians, and supporters about what recovery means–or whether the term is preferred relative to other terms (e.g., healing or remission). For decades, researchers have been working to establish a consensus definition of ‘recovery’ from an ED [28–30], but such a definition remains elusive. Increasingly, questions are emerging about the potential outcomes of a universalised definition of ED recovery when applied to a phenomenon that shows up differently depending on each individual’s identities and experiences [31]. People with lived and living experiences may differ in their perspectives about what recovery ‘is’ [32] compared to those treating them [33, 34]. Further, ‘objective’ criteria for determining recovery [35], such as symptom remission, may not fully map onto ‘subjective’ experiences of recovery [36, 37].

Questions remain about who standardising recovery definitions will most benefit. On one hand, distinguishing a set of criteria to delineate recovery can help to improve outcome reporting for treatment programs, increasing transparency and comparability of results [28, 38]. Greater consensus on the definitions of recovery, its assessment, and outcome measures may help determine a standard for the data that services and studies collect [39–41]. On the other hand, it is possible that by focusing on sameness rather than difference, the individual nuances of experienced recovery may be drawn into a singular ‘way’ of ‘being recovered’ that may be inaccessible to some [26, 31, 42]. Importantly, inaccessible standards of recovery may disincentivise engaging in treatment or initiating behavioural change by further entrenching feelings of hopelessness and futility in some individuals with EDs.

Healthcare systems and the costs and reimbursement for ED treatment vary significantly nationally and internationally, which affects treatment access, treatment outcomes, and conceptualisations of recovery outcomes published in ED literature [43–45]. The US context is overrepresented in ED research, meaning research occurring within this country’s non-single-payer healthcare system predominates. In the USA, healthcare coverage is not guaranteed, and ED treatment is accessed primarily through private health insurance and self-payment (16); ED treatment is limited for uninsured or publicly insured individuals [45, 46]. Differences in treatment access and structure internationally may contribute to mismatches between how EDs and recovery are conceptualised and the realities of the treatment landscapes many people with EDs face.

In countries with a national health service, access to ED treatment is often woefully scarce and disproportionately reserved for paediatric patients, individuals presenting at low body weights, or those demonstrating acute medical instability. Even when EDs are recognised [47], wait lists for funded services are often lengthy (up to 2–3 years for initial assessment and treatment) [48–51], resulting in some individuals sustaining longstanding ED durations by the time they are first able to enter care. In some countries with national health services (e.g., Canada, Switzerland, Spain, Singapore, Australia, the United Kingdom), a two-tiered system exists (particularly for mental healthcare), wherein those who can afford to pay out-of-pocket can bypass these wait lists and limited choice options to access private care. This system continuously disadvantages those unable to pay, likely further limiting equitable access to services. Available treatment types are also limited for those with public health coverage, with little to no choice about the *type* of services one may receive. Individuals who present at higher weights, those considered not critically ill enough or too critically ill, patients who previously had poor treatment outcomes, or those with physical or psychiatric co-occurring diagnoses may be viewed as too complex, denied support [10–12], and/or directed towards a palliative or hospice care pathway [52, 53]. These systemic restrictions [11, 16] can prevent access to timely and appropriate care and, in many cases, access to any care at all [46, 54] in ways that differ across national contexts [55–57]. These differences further complicate any attempts to create inclusive criteria for assessing ED recovery, staging, treatment responsiveness, or the potential for ‘terminality’ that are appropriate for individuals with diverse identities and experiences.

## Recovery as ‘normalcy’ or quality-of-Life?

For clinicians and many researchers, recovery is typically conceptualised as a combination of behavioural (e.g., symptom remission), cognitive (e.g., reduction of ED-related thoughts), and psychosocial (e.g., social connection) factors [27]. It is generally agreed that recovery is not determined by weight or nutritional restoration alone nor by symptom remission in the absence of other, broader changes [28]. Clinician conceptualisations tend to emphasise symptom remission and time-based criteria as a baseline upon which ‘functional recovery’ can be built [58]. Often, these definitions foreground a conceptualisation of recovery as absence of illness, where therapies are deemed to have eliminated pathology. This perspective prioritises “recovery from,” with medicalised criteria leading as primary aims; this can be contrasted with a recovery model orientation [59] which emphasises individually-determined criteria for recovery and the idea of “recovery in” [60]. A recovery model orientation has been proposed to resonate with EDs and in particular AN [61]. The model is even written into standards for clinical treatment such as *The Royal Australian and New Zealand College of Psychiatrists Clinical Practice Guidelines for the Treatment of Eating Disorders*; however, this encoding has not necessarily resulted in a greater degree of shared understanding of how recovery is tied into people’s lives more broadly [62]. As people explore and narrate their experiences with and through EDs, they may reclaim or reconceptualise their identities–and the degree to which recovery features as key–over time [63].

The idea of a ‘return to normalcy’ or the emergence of ‘normalcy’ is prominent in many clinical/healthcare provider accounts of recovery. While some do not problematise the idea of normalcy, others question what ‘normal eating’ or ‘normal body image’ would look like in a world that holds profoundly anti-fat and diet-culture-oriented ideologies [64]. Existing expectations for ‘full recovery’ often presume attaining either a completely intuitive and positive relationship to food and body unaffected by ubiquitous cultural forms of oppression, or engaging in ‘normative’ levels of dieting and restriction in line with cultural expectations that do not tip over into ‘disorder’ [65]. Again, there is an emphasis here on reaching a pre-illness state absent of pathology, which may or may not resonate with individual orientations to recovery.

Clinicians and patients may differ in their perspectives on recovery, including what is regarded as ‘treatment success’ [29]. It should be noted that while some studies reflect multiple recovery perspectives (i.e., those with lived experience and clinicians), these perspectives are typically isolated from one another and rarely compared [66]. Further, despite calls to construct transdiagnostic definitions of recovery [27, 28], many studies exploring lived experiences of recovery have focused specifically on the experiences of those diagnosed with anorexia nervosa (AN). This trend has begun to shift recently toward the inclusion of varied ED diagnoses and the experiences of those who have not been formally diagnosed. In addition to differences in defining and communicating about recovery *between* groups, there are differences *amongst* people within a particular group – for instance, *clinicians* may differ in how they frame recovery [64]. These differences may be partly informed by the populations with whom clinicians regularly work (e.g., adolescents vs. adults), the milieu in which they work (e.g., community vs. intensive settings), and the training they received. In studies assessing lived experience perspectives, there appear to be differences between those who do and do not experience formal treatment for EDs both in terms of how they *talk about* EDs and recovery [67, 68] and potentially how they *experience* EDs. Social narratives are influential in shaping assumptions about ED presentation and experiences in treatment, where the ED ‘stereotype’ [46, 69] is particularly salient and may play a role in how some individuals identify with aspects of recovery and/or illness. Quality-of-life is often a central concern for those experiencing what becomes known as ‘recovery’ [70], though it is not always included in clinical remission perspectives. Indeed, people in recovery may articulate a high subjective quality-of-life even without full symptom remission [38]. A quality-of-life focus enables a more personalised perspective on what recovery might mean to the individual, consistent with a non-linear imagining of recovery. Looking beyond full symptom remission allows exploration of other factors that might promote and sustain recovery [30].

## What constitutes a longstanding eating disorder?

In addition to the lack of consensus on definitions of recovery, defining and conceptualising ‘stages’ of EDs (i.e., categorising EDs based on illness duration, past response to treatment, or clinical impairment; [71]), particularly AN, remains a source of disagreement among clinicians, researchers, and people with lived experience [1, 72]. In determining what constitutes a longstanding ED, a contrast is typically drawn between individuals who respond ‘adequately’ to one or a small number of treatment interventions within a few years (response typically defined as remitting symptoms and/or weight restoration) and individuals who either do not complete such interventions or do not achieve lasting improvement from them [1].

The construct of a longstanding ED (particularly the framing of ‘SE-ED or ‘SE-AN’ as ‘distinct’ patient populations) necessitates an opposing category of a ‘transient’ [73] or a ‘shorter’ term ED [74]. However, non-longstanding EDs are generally treated as an unspoken default and rarely given an explicit descriptor. This nosological consideration is further obfuscated by the primary focus on (low-weight) AN in many considerations of ‘severe’ and long-lasting EDs, although other EDs may also have lengthy durations and result in severe impairment. Whether longstanding EDs are the exception or the rule is also a source of disagreement between researchers, with clinicians and researchers presenting conflicting claims and evidence. The originating authors of the proposed ‘terminal AN’ diagnostic construct [4–6] have argued that “The vast majority of individuals with AN of all ages and chronicity will fully recover, and this should always be the initial goal” [4, p. 8], later describing ‘SE-AN’ as a “well-recognised subset” comprising 20% of AN patients [75, p. 8]. What constitutes full recovery in this context is not specified. In contrast, other researchers have described EDs as ‘chronic conditions’ [76], stating that a minority of individuals ‘fully recover’ [74] based on a combination of symptomatic, clinical, and subjective recovery.

Robison et al. [25] recently analysed a retrospective cohort of individuals with AN in a higher level of care (HLOC) from admission to discharge who met the first three criteria for ‘terminal AN’ outlined by Gaudiani et al. [6] (i.e., (a) AN diagnosis, (b) age 30 or older, (c) previously participated in high-quality care), and a subset of patients who also met a proxy index of the fourth criterion: (d) clear, consistent determination by a patient with decision-making capacity that additional treatment would be futile, knowing death will result [6] (for this study, patients endorsed desire for death in a self-report measure) [25]. The patients who met the proposed criteria for ‘terminal’ AN, including those in the subcategory, did not demonstrate a progressive, inevitable declining course of illness leading to death [25]. Furthermore, the “terminal” AN (including those who met the proxy for the fourth criterion) and “not terminal” AN groups were heterogeneous and did not significantly differ in physiological status and psychological self-report measures at admission and discharge. An overall trend of improvement across physiological and self-report measures, provided some empirical evidence against the ‘terminal AN’ diagnosis [25].

Additionally, a transdiagnostic and disorder-specific systematic review and meta-analysis [77] analysed recovery outcomes in people with EDs. Recovery was defined by the absence of ED behaviours. In all EDs pooled together, the recovery rate was 42% at < 2 years, 43% at 2 to < 4 years, 54% at 4 to < 6 years, 59% at 6 to < 8 years, 64% at 8 to < 10 years, and 67% at ≥ 10 years [77]. In pooled EDs, self-injurious behaviours were associated with lower recovery rates [77], indicating an important co-occurring need that may contribute to complexity and a longstanding course. Overall chronicity (defined here as continued presence of an ED diagnosis) occurred in 25%, with no significant difference between ED-groups, and mortality occurred in 0.4% of people with no significant ED-group difference [77]. Notably, for individuals with AN, lower rates of recovery and higher mortality were correlated with a treatment waiting list [77]. Collectively, these findings highlight problems with the proposed ‘terminal AN’ diagnosis; as chronicity is not unique to those diagnosed with AN, potential for ED recovery is influenced by co-occurring conditions, and a uniquely ‘terminal’ stage of AN is not indicated.

Proposed illness durations for longstanding EDs vary widely, ranging from a minimum of three to 10 years or more of consecutive illness duration [2, 78]. However, some data suggest that the average *cumulative* illness duration for people with EDs falls within these proposed ranges [79, 80], and as stated by Gutiérrez and Carrera [81], “most adult patients belong to this category” [81, p.2]. Another unifying theme among proposals for the definition of a longstanding ED includes a lack of response to treatment [1]. However, treatment response and sustainability of improvements can be difficult to evaluate comprehensively, particularly due to the exclusion of underrepresented ED populations captured in demographic data [82]. Among individuals who access treatment, many discharge early (patient-initiated discharge) for reasons including dissatisfaction with services [83], low perceived efficacy [84], mistrust, therapeutic rupture [85], misalignment with treatment procedures or focus [86], time on waitlists, financial limitations, inadequate insurance coverage [87], or family responsibilities [88, 89], or are discharged early from treatment by providers (clinician-initiated discharge) [90, 91]. These differences in the reasons for premature ending of treatment may or may not be captured in data collection.

Evaluation of treatment outcomes and recovery research may also be complicated by commonly utilised ED measures that may not measure the same symptoms (low overlap and high heterogeneity [40]). Many individuals with EDs do not access treatment or have already sustained an ED duration that is considered longstanding by the time they first receive treatment [92], limiting the scope of which individuals and stages of illness research conducted in treatment settings can capture. As mentioned earlier, in single-payer healthcare systems, waiting lists for treatment may be as much as 2–3 years long (overlapping with some proposed minimum illness durations for ‘SE-ED’ or ‘SE-AN’ criteria; [78]), further underscoring that these designations may manifest as an iatrogenic product of healthcare constraints versus an organic manifestation of an individual’s particular biology or psychology predisposing them to an intractable ED presentation. Due to the lack of empirical data or professional consensus to support a cohesive understanding of a longstanding ED [78], a separation between a longstanding ED and a non-longstanding ED (rather than a spectrum perspective of durations) is questionable in its clinical utility.

## Iatrogenic harm - individual, clinician, and system impacts

Experiences of iatrogenic harm (unintentional physical, mental, or emotional illness or injury acquired by experiences in medical care) may impact individuals while seeking treatment [93–97], and should be considered a factor that may contribute to the progression into or maintenance of a longstanding ED. Iatrogenic harm may also occur via narratives in ED treatment and research, such as presenting recovery in an idealistic way or describing EDs as naturally ‘treatment resistant’ [98, 99]. Iatrogenic harm experienced in ED treatment, provider expressions of hopelessness about a patient’s ability to heal, and a decreased quality-of-life can make living with the ED feel more tolerable than recovery [12, 16]. Traumatic inpatient experiences have been described as destroying desire for recovery and instead, “putting the disease at my core” [100, p.171]. A vicious cycle experienced by some individuals in which the cyclical nature of EDs, iatrogenic harm, trauma, prolonged illness, and co-occurring psychiatric diagnoses can compound and exacerbate one another has been described by multiple authors with lived experience [7, 10, 12, 16, 17]. In turn, this cycle may decrease the chance for hopefulness, make ED behaviours more difficult to change, thereby contributing to ED duration.

The experience of seeking and receiving ED treatment can be fraught with contradictions, uncertainty, and loss of autonomy. Individuals with EDs may be at risk of being subject to involuntary treatment, pressured to accept ‘voluntary’ treatment under coercion to maintain greater freedoms that may be afforded with voluntary patient status [101, 102], or be paradoxically refused treatment due to the perceived intractability or severity of their ED. ED treatment programs or clinicians often use authoritarian treatment protocols; for example, the use of ultimatums and pseudo-contracts or ‘contingency contracts’ as a method of coercing individuals into accepting a HLOC and as a strategy for setting expectations of treatment reward, punishment, and behavioural change [6, 103, 104]. People with EDs are inordinately subjected to coercive or compulsive treatment methods [103] such as involuntary nasogastric tube feeding under restraint (actual or threatened), seclusion, and physical, mechanical, and/or chemical restraint [105, 106]. ED treatment may encourage patients to develop an agentic sense of self, utilise assertiveness skills, and develop greater independence while simultaneously punishing displays of agency that challenge or question treatment protocols (such as operant conditioning methods) [12, 107, 108]. This creates an orientation where patients are expected to comply with clinical ‘authority’ [107] and power exercised over their bodies, behaviour, and treatment decisions [12, 109]. Individuals may be prematurely discharged from services while still seeking treatment if treatment providers perceive them as not improving quickly enough, being ‘non-compliant,’ or presenting as ‘too complex’ [6, 10, 12].

Although the use of coercive and compulsory methods may be altruistically driven to preserve life [110–112] and induce behavioural recovery for some, these experiences can be traumatising, particularly as many individuals with EDs have or are suspected to have higher rates of neurodivergence [113], and do have higher rates of co-occurring diagnoses and trauma [114]. These negative experiences may influence future avoidance and distrust of ED treatment [115, 116]. Some individuals (but not all; [110]) who are treated involuntarily report retrospective gratitude or other benefits for involuntary treatment [111, 117, 118]. For some, this gratitude may coexist with having experienced involuntary treatment as traumatic, abusive, and degrading [12, 111, 115]. Additionally, use of restraint, confinement, and coercive methods may lead to substantial trauma or physical injury to the individuals subjected to them [12, 111], and also result in moral and physical injuries [119, 120], betrayal trauma, compassion fatigue [121, 122], and hopelessness [123] for clinicians involved in their administration. These impacts are important to consider in the conceptualisation and treatment of longstanding EDs, as clinician countertransference (i.e., impacts from participation in compulsory treatment) may affect the prognosis and valuations patients are given, including perceived ‘terminality’. Clinicians can feel traumatised by the complex acuity of hospitalised patients and may receive inadequate support for coping with their roles [12, 119]. These provider influences and attitudes may also have important impacts on the sense of futility, prognoses, and care pathways for individuals with EDs including perceived ‘terminality’.

Even when involuntary treatment occurs, compassion, the establishment of trust [121, 124], and as much collaboration as possible can minimise the risk and severity of trauma [125, 126]. An example of promoting autonomy for individuals with a longstanding ED is the provision of opportunities for supported decision-making processes [121], such as developing documents that outline wishes for treatment (i.e., Advance Health Directives and Ulysses Contracts) [127, 128] in jurisdictions where available. People with EDs may also be subjected to various dehumanising, stereotyping, and harmful judgments that bias clinicians and reduce the ability of family members and other social contacts to provide effective recovery-oriented support. For example, people with EDs can be viewed as fragile, childlike, manipulative, wilful [107, 129] and personally responsible for their illness and outcomes [130, 131]. Compared to individuals with other psychiatric or physical conditions, public attitudes toward individuals with EDs are more likely to be stigmatising, and at times, involve unique features of stigma, such as admiration and envy [129, 132]. Within both clinical and family settings, individuals with AN especially may paradoxically be both subjected to negative stereotypes and simultaneously have attributes of their AN romanticised and venerated (e.g., having extreme ‘willpower’) [133, 134], furthering the tendency to characterise the ED as a positive or sole source of self-concept [135, 136] and perpetuating misunderstandings about the reality of life with AN.

Individuals with EDs can also be negatively impacted by stigmatising narratives within the literature and clinical contexts, such as the frequent suggestion that EDs/people with EDs are ‘treatment resistant’ [98, 99] and ‘difficult to treat’ [107, 137], potentially biassing new clinicians into believing people with EDs inherently resist treatment [138, 139]. Such attitudes and semantic choices blame ED patients for poor treatment outcomes, implying an agentic failure to be treatable rather than a failure of current treatments to effectively treat them. These narratives also interrelate with critical power dynamics [85, 100, 108], and may consequently lead to or compound feelings of worthlessness and isolation [140], as well as relational ruptures [109, 110] iatrogenic harm, and response to or lack of response to iatrogenic harm [12, 141, 142].

Clinician-patient shared decision-making and co-produced treatment goal-setting between clinicians, patients, and their loved ones [83, 85] has been shown [111] to improve a sense of autonomy, balance power dynamics, and reduce the risk of premature termination of treatment. Rebuilding communication and the therapeutic alliance [141–143] may mitigate treatment avoidance [144–146] and in turn limit the perception that treatment is ‘futile’ for individuals who lose trust in treatment, do not respond to treatment in a way that is expected, and/or prematurely discontinue services [147].

## When is ED treatment futile, and who decides?

In the context of EDs, the concept of ‘futility’ has been applied to individuals believed to be unlikely to benefit from additional care [148], although clinicians generally acknowledge it is difficult to deem someone entirely ‘incurable.’ The concept of treatment futility is complex due to the limited effectiveness of available ED treatments [149, 150], financial and geographic limitations [11, 16] and differing conceptualisations and expressions of EDs cross-culturally [151–154] which impedes access to treatment that may be medically or psychologically indicated.

Concerningly, ‘treatment resistant’ is applied almost indiscriminately to individuals with EDs who are viewed as unresponsive to the psychotherapeutic and behavioural interventions they receive [98, 137, 139]. In contrast to EDs, ‘treatment resistance’ for other psychiatric disorders, such as major depressive disorder, bipolar disorder, and schizophrenia, is characterised by having limited or no responses to psychiatric medications or other interventions that typically have a higher efficacy rate [155]. A ‘palliative psychiatry’ approach to psychiatric treatment focuses on quality of life of individuals with ‘severe persistent mental illness’ (SPMI) but does not strive to achieve complete “disease remission” [156, p.2]. Trachsel et al. [156] suggests a ‘staging’ model to mental illness may be useful in identifying when psychiatric treatment can switch from ‘curative’ goals, but they “would not call persons with SPMI ‘terminally ill’” [156, p. 3]. As previously stated, in contrast to other mental illnesses, individuals with EDs are often described as ‘treatment resistant’ even though efficacious treatments are lacking; and, notably, a ‘terminal’ stage of AN has already been proposed [6], although a uniquely ‘terminal’ stage of AN is currently unsubstantiated [22, 25, 77].

Understandings of treatment responsiveness/resistance are complicated by the fact that individuals can be reluctant to change their ED behaviours due to the utilisation of ED behaviours as coping mechanisms [133], potential alignment of personal values with the ED, and the sense of identity the ED may provide [127]. These functions of the ED and the enmeshment with the self may lead to fear and uncertainty about life without this vital part of life and sense of identity [157]. However, low motivation to change and being ‘treatment resistant’ are not synonymous [107, 135] and ‘treatment non-response’ and ‘lack of motivation’ discourse may erase individuals’ repeated attempts to recover and the difficulties and adversities they encounter [100, 108, 158–160].

The frequency of psychological co-occurring diagnoses and the psychological impact of chronic health conditions and complexity in individuals with EDs [161, 162] are entwined with conceptualisations of futility in EDs. Some existing models of care may not consider co-occurring psychological symptoms and impacts, which, if under-addressed or unresolved, may impede ED recovery. While treatment models have been developed to address EDs and co-occurring diagnoses [80, 163–167], many such approaches have not been integrated into mainstream treatment for EDs [46]. Widely used treatment models enforce inflexible ‘behavioural protocols’ and emphasise ‘focusing on the ED first’ at the expense of meeting the treatment needs of some individuals [80] who may otherwise benefit from approaches that address self-concept and embodiment (which can be disrupted and disembodied in individuals with EDs) [168]. This highlights the importance of treating ED and co-occurring conditions together, particularly as depressive symptoms associated with malnutrition may improve after initial medical stabilisation [169, 170]. Provider uncertainty and inexperience in managing and treating co-occurring conditions may contribute to vulnerability, inadequacy, and despair [109, 171], and these exacerbated feelings may subsequently affect the clinician’s perceived prospect of their patient’s chances to recover and/or the individual’s belief in their own ability to recover.

The impact of treatment that does not meet an individual’s needs can be significant. Receiving treatment multiple times without substantial improvement and/or poor therapeutic delivery can lead to helplessness and hopelessness around therapeutic response and recovery [162, 172, 173]. Conversely, providing narratives of hope across ED literature, clinical practice, and prevention strategies can lead to more treatment engagement [174, 175]. Critically, multiple accounts from people with longstanding EDs report that *hope* is integral [7, 10, 12, 17, 157] across treatment methods or modalities [176], and across durations of ED, but especially for those with a longstanding ED [177]. Conceptualisations of futility and descriptions of a proposed ‘terminal’ stage of AN [6, 75, 178] frequently include experiences of iatrogenic harm, complex co-occurring diagnoses, not-responding to initial treatment/s, and hopelessness for recovery - rather than denoting a state of inevitable, irreversible illness, decline, and death. Rather, these characteristics describe an ED treatment system with considerable gaps when it comes to meeting the needs of individuals with EDs, including: failing to understand and respond to factors such as inequity in care [16], treatment gaps (e.g., lack of accessibility, ED competent outpatient clinicians, collaboration between services, and integrated care), iatrogenic harm and lack of repair work [12, 53, 141, 142, 150], and clinician-patient expressions of hopelessness in likelihood of recovery [12].

There can still be opportunities for hope, repair, and healing for individuals long underserved and harmed by these treatment systems. June Alexander (who has recovered after more than 40 years of ED; [14]) explains what hopefulness may be for someone with a longstanding ED: “I find the word ‘recovery’ is not always appropriate for someone who has reconnected with their healthy self after decades with anorexia. Those decades with anorexia do not miraculously disappear…we cannot ‘recover’ who we were prior to the illness… I prefer the term, ‘ongoing healing’” [14, p.3]. Critically, such ‘ongoing healing’ in people with longstanding EDs may need to encompass healing not only from the ED itself but also from iatrogenesis incurred during previous treatment experiences. Attentiveness to both of these components has rarely been considered within the ED treatment literature, despite their importance, especially in assessments of treatment futility and intractable illness.

## Conceptualisations of terminality in eating disorders: medical and psychological considerations

Definitions of terminal illness are, in general, ambiguous, with varying clinical and research criteria used to conceptualise this state [179]. Unifying themes, however, have been found to include an irreversible disease with limited survival duration (duration varies by definition, e.g., less than 24 months, 12 months, 9 months, 6 months, and 3 months; [179]). McCartney and Trau (1990) suggest that a terminal illness should be defined as a condition that, “to a reasonable degree of certainty, there can be no restoration of health, and which, absent artificial life-prolonging procedures, will inevitably lead to natural death” [180, p.438]. Notably, these definitions may be applied to illnesses where prognosis – while never certain – can be determined with greater certainty than is possible with EDs [22, 23, 25, 77]. Additionally, determining ‘restoration of health’ is challenging given the aforementioned differences in conceptualising recovery for EDs.

Historically and presently, most ED research has focused on AN – as have discussions about ‘terminality.’ Even in this context, however, what it means to ‘restore health’ depends on the context in which ‘health’ is being defined – and whose perspective is foregrounded. EDs, particularly AN, have high mortality [181]. However, the medical sequelae of AN are incomparable to conditions typically regarded as terminal (e.g., amyotrophic lateral sclerosis, some cancers; [22, 24]), which have “clear, objective parameters” [24, para.5] that may lead to death. Hypoglycemia, electrolyte imbalances, and cardiac arrhythmias are among the medical complications of AN, which can be fatal [182, 183] but are often abrupt, difficult to predict [184] and can be reversible with medical care and nutrition [25, 185]. The proposed conceptualisation of ‘terminality’ by Gaudiani et al. [6] is essentially based on a psychological, emotional, and even existential terminal state (provided that some physical and logistical criteria are met) rather than a physical state of irreversible, moribund decline. They argue, “Very specifically, to move toward a designation of ‘terminal AN’, an individual must express consistently that they can no longer live with their disease and will no longer maintain a minimum nutritional intake needed to support life” [6, p.13]. To some extent, this is a tautology: an individual would be considered to have ‘terminal AN’ because they express consistently that their AN is terminal. June Alexander delineates an important distinction between the mortality of AN and a hypothetical terminal stage: “Yes, there will be deaths—from organ or other physical failure, from suicide—there is only so much a body can take—but to ‘predict’ a termination of a life wracked with AN by placing a label on suspect patients would be fraught with dangerous risk of misinterpretation” [14, p. 13–14].

In EDs, chronicity or possible ‘terminality’ must not be conflated with or defined by low motivation for treatment previously experienced as ineffective or traumatic, co-occurring psychiatric disorders, decreased quality-of-life, suicidality, and untreated malnutrition [186–188]. Many of these feelings or states are common among people with EDs regardless of illness duration or ED diagnosis [173, 189–191], and some are regarded as ‘hallmarks’ of an ED [192]. People with EDs may have a relationship to death broadly and death from an ED in particular [12, 193–195] that may be unfathomable to non-ED populations, and this must be appropriately understood and factored into any assessment of or criteria for ED ‘terminality.’ People with EDs commonly express feelings of treatment unworthiness [196, 197], guilt for the impact of their ED on others [12, 198] and endorsement of the necessity of presenting in a physical state of extreme severity to feel their suffering is valid and deserving of help [188, 197, 199]. Feelings of pervasive unworthiness may also present with hopelessness, passive suicidality, and the belief that death would be preferable for themselves or their family rather than suicide, provided that their death occurs through the consequences of starvation [12, 200, 201]. Individuals with EDs may perceive death from starvation as a way to: express their wish to disappear [168] or their belief that they do not have ‘any right to live’ [200, p. 561], self-harm, and self-punish, die prematurely, provide a less painful death [200–203], and/or make their death ‘unnoticeable’ or less impactful to people close to them [12]. Furthermore, EDs can be a process of (dis)embodiment, where one loses their ‘true self’ to their ED and death can be both an escape from themselves and an escape from the “torturing thoughts… of [the] eating disorder” [168, p. 9].

These beliefs stand to make the proposed definition of ‘terminal AN’ [4, 6] highly ego-syntonic in some ED populations, with this designation potentially reinforcing the possibility of death from the ED as simultaneously a hard-earned reward and a deserved punishment. Past concerns that the introduction of diagnostic criteria for ‘terminal AN’ may lead people with EDs to view this construct and the option of Medical Aid in Dying (MAiD) “as a logical appealing solution to their suffering” [20, p.2] are not unrealistic. Several lived experience perspectives have echoed this sentiment that the suggestion or option of MAiD for individuals considered to have ‘terminal AN’ can “create a new experience in shaping how an individual thinks and relates to their experience, the feelings and responses of others, choices and outcomes” [12, p.3] and “may come to represent an aspiration for many who believe that their suffering and autonomy will only be respected if they can ‘succeed’” [16, p.6] at meeting Gaudiani et al’s. four criteria. Author LC in Downs et al. [11] writes, “[the terminal AN construct] would have been further ‘proof’ that I was never going to get better, that I should in fact die. I may even be provided with assistance in the form of medical aid in dying to help me finish this existence” [11, p.149]. A failure to recognise the psychological impact of being considered to have ‘terminal AN’ and the opportunity to use MAiD minimises the often desired and valued nature that having [204] or even dying from AN may have for some people [194, 200, 205]. This is particularly relevant as some individuals experience the ED as a salient aspect of identity, which is especially characteristic of a longstanding ED [157, 173, 206]. Individuals may perceive a diagnosis of AN as a ‘life sentence’, providing them with a label that undermines hope and attacks their sense of self-worth while simultaneously offering an identity they may wish to defend against losing [95, 168].

MAiD has also recently been proposed as an option for individuals with AN who are determined to have decisional capacity to decide further treatment is futile (i.e. criteria four for ‘terminal AN’) and meet other specified criteria [6]. Nevertheless, many scholars have described ethical [24, 207–209] contextual [19, 24], and methodological [20, 210–213] difficulties in assessing decision-making capacity for individuals with AN. Gaudiani et al. [6]. , propose that individuals with AN who meet the authors’ criteria for ‘terminal AN’ and are experiencing ‘intractable suffering’ would have the ability to: avoid a protracted death from malnutrition through access to MAiD, be relieved of the mandate to endure additional courses of treatment previously experienced as traumatic and ineffective, and control the timing of their death. However, advocates for the use of MAiD for EDs may not appreciate the reality that justifying a ‘terminal’ ‘stage’ in AN and MAiD pathways can funnel ambivalent individuals into a ‘death track’ [12, p.12] which stands to both cement psychological orientation towards death and limit further access to recovery-oriented care pathways.

In studies of longstanding AN, individuals have described a loss of agency wherein their AN became a ‘puppet master’, ‘conductor’ and a ‘sniper’ that has ‘taken over’ [168, 214]. One individual described that when her AN was strongest, “that’s when I wanted to die… I was a slave, I wasn’t in charge anymore, didn’t dare to stand up, was afraid of everything” [214, p.4]. These individuals frequently experienced “complex, emotional and changeable relationships with healthcare professionals and people in their social circle, as well as with the (AN) itself [214, p. 8],” and their wish to break free of it [168, 214], highlighting the importance of fluctuating states in longstanding AN. The impact of a ‘terminal’ ED ‘stage’ or ‘phase’ for an individual precludes the potential for common fluctuations in despair, endorsed feelings of wishing for death, and future orientation to sustain the possibility of healing and improvement [12, 25, 215]. Juxtaposing a peaceful death through MAiD with severe forms of iatrogenic harm experienced in ED treatment, as many authors have done [4, 6, 216–218], presents a false dichotomy between compassion for the person and treatment of the ED, and risks providing dangerous justification.

## Towards an individualised conceptualization of recovery: what becomes possible?

‘Terminality’ concepts in EDs exist inherently in tension with conceptualisations of recovery, as the determination that a patient has reached a ‘stage’ of AN (or another ED) constituting a terminal illness precludes the possibility of recovery. Operational definitions of recovery are, therefore, critical to assessing the validity of either ‘terminality’ in EDs generally or the perception of a particular individual’s prognosis as ‘terminal.’ As we discussed earlier in this paper, clinical perspectives and definitions of recovery can be inconsistent and out of sync with those with lived experience [32–34]. We recognise that a focus on harm-reduction, person-centred care, and quality-of-life will not eliminate deaths from EDs, nor will they necessarily be able to provide meaningful improvement for every person currently experiencing a longstanding ED. This paper is an overarching consideration of some of the conceptual, experiential and nosological factors that we believe have been under-considered in existing ED literature on ‘terminality’ and does not seek to provide universal or individual solutions.

Measuring up to rigid and idealistic definitions of recovery may indeed be unlikely for some individuals; however, a more individualised and person-centred approach to healing can broaden the conceptual territory in which alternatives to ‘terminality’ can be explored. For example, autistic people [219, 220], people with gastroparesis [221, 222], people with sensory processing differences [219], and individuals facing food insecurity [223, 224] may or may not be physically able to eat in an intuitive way imagined in some conceptualisations of recovery. Likewise, people with insulin-dependent diabetes can embody recovery in a way that works for them and enhances their overall quality-of-life, but their ways of recovering, particularly around food, may look different than those who do not have diabetes [225–227]. These factors can impede normative expectations of participation in social and professional activities, frequently considered evidence of and promised to be achievable in recovery [30].

Regarding harm-reduction (also referred to as ‘harm-minimisation’), Yager et al. [4]. , argue that harm-reduction approaches should only be considered after a prolonged illness and the determination that ‘full recovery’ is ‘unlikely’. However, minimising harm by focusing on personal goals and values [188, 207, 228] from the start of treatment may mitigate feelings of inadequacy, hopelessness, and potential for chronicity. Given that many treatment paradigms position the presence of any ED thoughts or behaviours as a detriment to quality-of-life and full personal development [30, 36, 37], considerations of how to maximise quality-of-life and personal development even if some level of ED symptoms remain can reframe harm-reduction away from being a last resort.

For some individuals with EDs underserved by the ‘full-recovery’ paradigm, holding that EDs can be disabling while still affirming personhood and value alongside the presence of impairment from the ED may increase an experience of greater hope, autonomy, and efficacy [177, 188, 207]. Investing in and celebrating quality-of-life while currently living with an ED rather than in the theoretical ‘after’/remission from an ED may appear counter to commonly established narratives of recovery [176, 207] - however, harm-reduction may present a more realistic and accessible option in the short and long-term for many people [12, 188, 207]. To be clear, we frame harm-reduction not as a precursor to palliative or hospice care only after recovery-oriented treatment has been deemed futile, but as a way in which ED treatment may better facilitate opportunities for recovery from the start for some individuals. One possible way of approaching treatment and harm-reduction differently is to consider the lens of the social model of disability, which is under-explored in the context of EDs. The social model of disability invites us to consider how the world is dis/abling rather than situating the ‘problem’ of disability within an individual [229]. Garland-Thomson [230] engaged with ‘fitting’ and ‘misfitting’ to explain how bodies and worlds are interlinked; consequently, social and material conditions can arise in how bodies come into contact with the world around them–which may not be configured to accommodate their needs.

In the context of EDs, a social disability lens can reorient the focus away from fixing a perceived deficit within the individual, which is preventing them from accessing certain ways of being in the world. Instead, the emphasis is on exploring what kinds of *social and systemic changes* might enable greater access and belonging–and, ultimately, a ‘recovery’ that ‘fits’ better for the person. For example, some individuals’ recovery or ED stability could include longer-term use of oral supplements or a feeding tube to meet nutritional needs. While longer-term use of supplements or tube feedings may not resemble the ‘recovered’ or ‘normal relationship’ with food outlined by some treatment paradigms, it can enable some individuals to access a meaningful quality-of-life, meet nutritional needs, and maintain medical stability [12, 207]. Individuals with EDs often defy the odds of recovery or a reduction in ED symptoms predicted by their providers and themselves [10, 12–14, 17, 18, 25, 231–235]. Furthermore, over the course of an ED, individuals commonly experience fluctuations in symptoms [12, 52, 178, 232, 236, 237], insight into the ED [238, 239] as well as depression, suicidality, and endorsement of death wishes [12, 25, 240]. Additionally, people can experience behavioural recovery and/or a personalised sense of recovery after decades of illness [14, 15, 18, 52, 76, 79, 188, 214, 231, 233, 240–242]. Collectively, we have highlighted the challenges and limitations in the operationalisation and validity of conceptualising and defining longstanding EDs and ‘terminality’, as well as potential consequences with current definitions.

## Conclusion

‘Terminality’ in EDs as an operationalisable concept is predicated on conceptualisations of recovery, treatment non-responsiveness/futility, and thorough exhaustion of supposedly adequate and available treatment options, all of which we argue are inconsistently and inadequately accounted for in existing research and clinical contexts. Proposing new and consequential categories of ED, particularly in relation to MAiD, introduces many substantial risks and unanswered questions. A greater focus on individualised conceptualisations of healing, alternate approaches to symptom and behaviour management, and investment in overall quality-of-life can offer more salient and hopeful potentialities to individuals where common understandings of ‘full’ recovery are inaccessible or who are at risk of being declared chronic, untreatable, or ‘terminal.’ As past assertions of ‘terminal AN’ have required patient endorsement of hopelessness and intractable suffering [4, 6] rather than specific medical risks or sequelae [25], considerations of how alternate and individualised conceptualisations of recovery may enable increased hope, resilience, and empowerment for individuals with EDs should not be overlooked.

## Data Availability

No datasets were generated or analysed during the current study.

## Footnotes

1 We use the term longstanding ED. As ‘SE-AN’ and ‘SE-ED’ are used inconsistently in the literature and we have multiple concerns about these terms, including that they may both stigmatise individuals with longstanding EDs and instil the belief that some people with EDs are not ill ‘enough’ (i.e., ‘not severe’) [to warrant these terms, concern associated with them, and/or triage of limited resources], we do not use these terms except in reference to their use in other articles.

2 In this article, we use ‘terminality’ and ‘terminal’ to refer to frameworks articulated by other authors that describe a hypothesised stage of ED (particularly anorexia nervosa) in which recovery or long-term survival is unlikely or impossible and death from the ED becomes inevitable. In our interrogation of this diagnostic premise, we do not question that EDs can be and are sometimes deadly, but do not consider this the same as EDs being sometimes ‘terminal.’ We therefore use ‘terminality’ or ‘terminal’ to describe this proposed addition to ED ‘staging’ frameworks but not to describe death or the possibility of death from an ED.

## Abbreviations

AN: Anorexia nervosa
CBT: Cognitive Behavioural Therapy
DSM-5: Diagnostic and Statistical Manual of Mental Health Disorders, fifth edition
EDs: Eating disorders
ED: Eating disorder
HLOC: Higher level of care
LGBTQA+: Lesbian, gay, bisexual, transgender, asexual
MAiD: Medical aid in dying
SE-AN: Severe and enduring anorexia nervosa
SE-ED: Severe and enduring eating disorders
SPMI: Severe and persistent mental illness
UK: United Kingdom
USA: United States of America
